# Women’s decision-making power and knowledge of prevention of mother to child transmission of HIV in sub-Saharan Africa

**DOI:** 10.1186/s12905-022-01691-4

**Published:** 2022-04-12

**Authors:** Betregiorgis Zegeye, Bright Opoku Ahinkorah, Edward Kwabena Ameyaw, Abdul-Aziz Seidu, Comfort Z. Olorunsaiye, Sanni Yaya

**Affiliations:** 1 HaSET Maternal and Child Health Research Program, Shewarobit Field Office, Shewarobit, Ethiopia; 2 School of Public Health, Faculty of Health, University of Technology Sydney, Ultimo, NSW Australia; 3 Department of Population and Health, College of Humanities and Legal Studies, University of Cape Coast, Cape Coast, Ghana; 4 Department of Public Health, Arcadia University, Glenside, PA USA; 5 School of International Development and Global Studies, Faculty of Social Sciences, University of Ottawa, 120 University Private, Ottawa, ON K1N 6N5 Canada; 6 The George Institute for Global Health, Imperial College London, London, UK

**Keywords:** Women, Decision making, PMTCT, Knowledge, Sub-Saharan Africa, Global health

## Abstract

**Background:**

Sub-Saharan Africa (SSA) bears the highest burden of Human Immunodeficiency Virus (HIV) in the world. Even though the prevention of mother to child transmission (PMTCT) programmme is one of the strategies to control the HIV pandemic, the uptake in SSA countries is low. Women’s decision-making power has a positive influence on health seeking behavior and uptake of several maternal health services. However, its relationship with knowledge of PMTCT services is understudied in SSA. Therefore, this study aimed to examine the association between women’s decision-making power and knowledge of PMTCT in 24 countries in SSA.

**Methods:**

Analysis of this study included data on 158,812 married women from the Demographic and Health Surveys of 24 sub-Saharan African countries conducted between 2010 and 2020. Using Stata version-14 software, bivariate and multivariable logistic regression analyses were conducted. The results were presented using adjusted odd ratios (aOR) with the corresponding 95% confidence intervals (CI).

**Results:**

In the pooled results, 69.5% (95% CI; 66.7–72.1%) of married women in the studied countries had knowledge of PMTCT, ranging from 13.9% (95% CI; 11.9–16.2%) in Comoros to 75.4% (95% CI; 73.7–76.9%) in Zimbabwe. Higher odds of PMTCT knowledge were seen among married women who had decision-making power compared to married women who had no decision-making power. Moreover, we found higher odds of PMTCT knowledge among married women with manual occupation, those in the richest households and those with 1–2 children compared to married women who were not working, from the poorest households, and those with no children, respectively.

**Conclusion:**

Women’s decision-making power had positive influence on PMTCT knowledge. To increase the coverage of PMTCT knowledge, policy makers and other stakeholders need to target ways to empower women through increasing women’s decision-making power. Moreover, creating employment opportunities and economic empowerment for women need to be considered, especially in countries with very low coverage of PMTCT knowledge.

## Background

Globally, slow progress has been seen in the reduction of new HIV infections; 23% decline from 2010 to 2019 (1). In 2019, an estimated average of 1.7 million (1.2 million to 2.2 million) new HIV infections were reported worldwide [1]. Sub-Saharan Africa (SSA) has the highest prevalence of HIV [1–3].

Maternal transmission of the virus (i.e., from an infected mother to the baby which can occur during pregnancy, delivery, and breastfeeding) accounts for most new HIV infections in children aged 0–14 years [1, 4]. Prevention of mother-to child transmission (PMTCT) programmes have numerous benefits for reproductive-aged women who are at risk of HIV or living with HIV, including preventing unwanted pregnancy and enabling them to keep up their health and prevent their babies from getting infected by HIV [1, 4].

Maternal transmission of HIV by a positive woman to her baby can be reduced to below 5% by using antiretroviral treatment (ART), from 15 to 45% transmission possibility without ART [1, 5]. Worldwide, because of PMTCT programmes, about 1.4 million HIV infections among children were prevented from 2010 to 2018 [1]. In Africa (Eastern and Southern) because of PMTCT services, the percentage of children who acquired HIV infection from their mothers has reduced from 18% in 2010 to 10% in 2017 [1, 6].

In SSA, uptake of PMTCT, including unmet need for contraception for HIV positive women is low [1]. For instance, ART coverage for pregnant women living with HIV was lower than 50% in Angola and was between 50 and 69% in four sub-Saharan African countries (Chad, Ghana, Ethiopia, and Democratic Republic of Congo) [1, 6].

Research shows that knowledge of PMTCT has positive influence on the uptake of PMTCT services [1, 3, 7, 8]. However, studies in sub-Saharan African countries show women’s PMTCT knowledge is low. For instance, a study in Tanzania shows that only 46% of women had adequate knowledge of mother to child transmission (MTCT) and PMTCT [9]. Other studies in Ghana [10] Ethiopia [8] and Cameroon [11] have demonstrated that only 10%, 22.4% and 23.7% of women, respectively, had PMTCT knowledge.

Several factors influence PMTCT knowledge among married women, including women’s socioeconomic status, occupation, place of residence, parity, exposure to mass media, and utilization of maternal health services [8, 12–15]. Evidence suggests that women’s decision-making power has positive influence on the utilization of many maternal healthcare services [16–19]. However, its influence on PMTCT knowledge is not well studied. Therefore, this study aimed to examine the association between women’s decision-making power and PMTCT knowledge in SSA. The findings from this study could contribute to policy development to enhance women’s decision-making power and to increase coverage of PMTCT knowledge in SSA.

## Methods

### Data source

We extracted data from the Demographic and Health Surveys (DHS) of 24 countries in SSA conducted between 2010 and 2020. The DHS are nationally representative cross-sectional surveys aimed at collecting data for monitoring demographic and several health indicators, including knowledge of PMTCT, across low-and middle-income countries [20]. The surveys are carried out with the financial and technical support of the United States Agency for International Development (USAID) and ICF International [21]. A two-stage stratified cluster sampling approach is applied. In the first stage, enumeration areas (EA) are selected from the sampling frame prepared in the most recent national population census using probability proportional to size (PPS) [22]. In the second stage, using systematic sampling technique, fixed numbers of households (usually 25–30 households) are selected from already selected EAs [22]. We selected 24 sub-Saharan African - countries based on the inclusion criteria: availability of the essential variables considered for this study as well as countries with DHS published between 2010 and 2020. We included 158,812 married women from the individual recode (IR) file since the independent variable is applicable only to married women [23–25]. The data are publicly available at: https://dhsprogram.com/data/available-datasets.cfm. More information about the list of included countries, year of survey and the corresponding samples is presented in Table 1 below.

**Table 1:** Year of survey for each studied countries and sampled population

Country	Year of survey	Sampled population	
		Weighted number	Weighted %
Angola	2015/16	5,123	3.23
Burkina Faso	2010	11,836	7.45
Benin	2017/18	4,271	2.69
Burundi	2016/17	9,161	5.77
Democratic Republic of Congo	2013/14	9,249	5.82
Cote d’Ivoire	2011/12	5,030	3.17
Cameroon	2018/19	7,068	4.45
Ethiopia	2016	7,495	4.72
Gabon	2012	4,272	2.69
Ghana	2014	4,480	2.82
Gambia	2013	6,024	3.79
Guinea	2018	4,552	2.87
Comoros	2012	2,379	1.50
Liberia	2019/20	3,703	2.33
Mali	2018	5,182	3.26
Malawi	2016/17	14,851	9.35
Rwanda	2014/15	6,837	4.31
SierraLeone	2019	7,555	4.76
Senegal	2010/11	7,653	4.82
Chad	2014/15	2,530	1.59
Togo	2013/14	5,447	3.43
Uganda	2016	11,112	7.00
Zambia	2018/19	7,137	4.49
Zimbabwe	2015	5,865	3.69
Total		**158,812**	**100.00**

Bold values indicate differentiate the variables

### Study variables

#### Outcome variable

The outcome variable for this study was knowledge of PMTCT among married women. In the DHS, women of reproductive age are asked four questions to measure their knowledge about PMTCT. These are: Can HIV be transmitted from a pregnant woman to her baby during pregnancy? Can HIV be transmitted from a pregnant woman to her baby during delivery? Can HIV be transmitted from a pregnant woman to her baby during breastfeeding? Can the risk of mother-to-child transmission reduce by taking special drugs? Women who responded affirmatively to all four questions were considered to have knowledge of PMTCT, and were coded as 1, otherwise they were considered as not having knowledge of PMTCT and were coded as 0. Respondents could also indicate “don’t know” which was treated as a lack of knowledge. Women who have not heard of HIV/AIDS are not asked these questions but were still included in the denominator; if they have not heard of HIV/AIDS they are assumed not to have this knowledge [25–27].

#### Explanatory variable

The explanatoryvariable was women’s decision-making power. In the DHS, to indirectly examine women’s empowerment, women are asked three decision-making questions. Who decides about your own health? Who decides to purchase large household expenses? Who decides when you want to visit family or relatives? Women who usually decided either alone or together with the husband on all three above-mentioned decision-making parameters were considered as empowered and were coded as 1, while those who indicated otherwise were considered as not empowered and were coded as 0 [24, 25].

### Covariates

By referring to the literature on factors associated with PMTCT [8–18, 23, 24, 26, 27], we included the following covariates: women’s age in years (15–19, 20–24, 25–29, 30–34, 35–39, 40–44, 45–49), women’s educational level (no formal education, primary school, secondary school, higher), husband educational level (no formal education, primary school, secondary school, higher), women’s occupation (not working, professional or technical or managerial, agricultural, manual, others), religion (Christian, Muslim, others, and no religion), place of residence (urban, rural), parity (0, 1–2, 3–4, 5+), media exposure (no, yes). With media exposure, we coded yes if the women read newspaper or listened radio or watching television, for at least less than once a week, otherwise we coded no.

Another included covariate was wealth index (poorest, poorer, middle, richer, richest). In DHS, wealth index is usually computed using durable goods, household characteristics and basic services following the methodology explained elsewhere [28], and we followed the same procedure. Other variables were barriers to accessing healthcare services, coded as “no” if the women had no big problems with any of the following four barriers; money needed for treatment, permission of husband to go to health facility, distance to health facility and not wanting to go alone to health facility and coded as “yes” if the women had a big problem with at least one of the four barriers.

### Statistical analyses

Using version14 of the Stata software, analysis for this study was carried out as follows. First, descriptive analysis including frequency distribution of respondents, overall PMTCT knowledge and its distribution across the explanatory variable and covariates was conducted. Then, chi-square test of independence was carried out to select variables that had significant association with PMTCT knowledge at p-value of 0.05 for bivariate logistic regression analysis. Subsequently, multicollinearity test was done using variance inflation factor (VIF) for all statistically significant variables from the bivariate logistic regression analysis, and we found no evidence of significant collinearity among the explanatory variables (Mean VIF = 2.46, Min VIF = 1.56, Max VIF = 6.14). Based on available evidence, mean VIF less than 10 is acceptable [29, 30]. Lastly, we carried out a bivariate and multivariable logistic regression analysis by entering selected explanatory variable/covariates into the binary logistic regression model. Model fitness was checked by Hosmer–Lemeshow test and confirmed that the model was fit (*P* = 0.1898). The results were presented using crude odds ratios (cOR) and adjusted odd ratios (aOR) at 95% confidence intervals (CI). To take care of the complex nature of the DHS data, we used the “svyset” command during the analysis and all three design elements such as weight, cluster and strata were taken into consideration. For preparation of this manuscript, we followed the guidelines for strengthening the reporting of observational studies in epidemiology (STROBE) [31].

### Ethical considerations

We used publicly available DHS data from DHS Program for the analysis in this study (available at: https://dhsprogram.com/data/available-datasets.cfm). Since the institution commissioned, funded, and managed the survey, further ethical clearance was not required. ICF international ensured that the protocol of the survey was compliant with U.S. Department of Health and Human Service regulations for the protection of human subjects. For more details related to ethical issues, readers can visit http://goo.gl/ny8T6X.

## Results

### Background characteristics of respondents

In total, 158,812 married women were included in the analysis for this study. Among them, 7.9% were 15–19 years of age. Approximately 15.9% of the respondents and 14.4% of their husbands had no formal education. About 20.6% of the participants were rural residents. Close to 66.4% of respondents had barriers to accessing healthcare services, including lack of money, distance to health facility, permission from husband, or not wanting to go alone. Approximately 67.2% of married women decided on all three of the decision-making parameters; their own health, purchasing large household expenses, and visiting family or relatives (Table 2).

**Table 2:** Knowledge of PMTCT among married women across explanatory/control variable/s: evidence from 24 sub-Saharan African  countries

Variables	Number (Weighted %)	Knowledge of PMTCT (Weighted %)		χ^2^, *P*-value
		No	Yes	
*Age in years*				χ^2^ = 44.22, *p* < 0.01
15–19	9677 (6.6)	42.9	57.1	
20–24	28,115 (19.8)	33.3	66.7	
25–29	34,039 (23.3)	26.9	73.1	
30–34	30,173 (17.8)	26.7	73.3	
35–39	25,257 (14.3)	29.4	70.6	
40–44	17,879 (11.6)	31.2	68.8	
45–49	13,185 (6.6)	33.7	66.3	
*Women’s educational level*				χ^2^ = 171.20, *p* < 0.001
No formal education	61,569 (15.9)	41.3	58.7	
Primary school	54,459 (37.6)	36.9	63.1	
Secondary school	36,708 (40.7)	21.7	78.3	
Higher	5580 (5.8)	20.5	79.5	
*Husband educational level*				χ^2^ = 151.71, *p* < 0.001
No formal education	54,797 (14.4)	37.5	62.5	
Primary school	44,344 (19.5)	43.5	56.5	
Secondary school	46,039 (55.7)	26.2	73.9	
Higher	12,160 (10.4)	19.6	80.4	
*Women occupation*				χ^2^ = 267.52, *p* < 0.001
Not working	38,332 (28.1)	28.3	71.7	
Professional or technical or managerial	6,356 (7.4)	22.8	77.2	
Agricultural	60,068 (17.0)	53.2	46.8	
Manual	12,210 (4.0)	18.4	81.6	
Others	41,268 (43.5)	25.4	74.6	
*Wealth index*				χ^2^ = 487.16, *p* < 0.001
Poorest	32,271 (9.1)	56.4	43.6	
Poor	32,157 (13.0)	53.6	46.4	
Middle	31,379 (22.0)	32.8	67.2	
Rich	30,543 (27.4)	23.6	76.4	
Richest	31,975 (28.5)	16.4	83.6	
*Parity*				χ^2^ = 27.98, *p* < 0.01
Zero	10,052 (3.4)	41.5	58.5	
1–2	49,247 (32.0)	27.4	72.6	
3–4	44,621 (30.3)	28.8	71.2	
5+	54,405 (34.3)	33.7	66.3	
Barriers to access healthcare				χ^2^ = 26.71, *p* < 0.01
No	54,376 (33.6)	25.8	74.2	
Yes	103,869 (66.4)	32.8	67.2	
Religion				χ^2^ = 3.91, *p* = 0.5948
Christian	95,283 (94.9)	30.2	69.8	
Muslim	56,586 (0.4)	33.2	66.8	
Others	3,893 (0.7)	33.5	66.5	
No religion	2,972 (4.1)	36.4	63.6	
Place of residence				χ^2^ = 321.07, *p* < 0.001
Urban	53,452 (79.4)	24.6	75.4	
Rural	104,873 (20.6)	53.1	46.9	
Mass media exposure				χ^2^ = 127.13, *p* < 0.001
No	61,784 (15.7)	47.3	52.7	
Yes	96,304 (84.3)	27.4	72.6	
Decision making				χ^2^ = 65.91, *p* < 0.001
No	88,505 (32.8)	37.9	62.1	
Yes	69,693 (67.2)	26.8	73.2	

### Knowledge of PMTCT

**Fig. 1:**
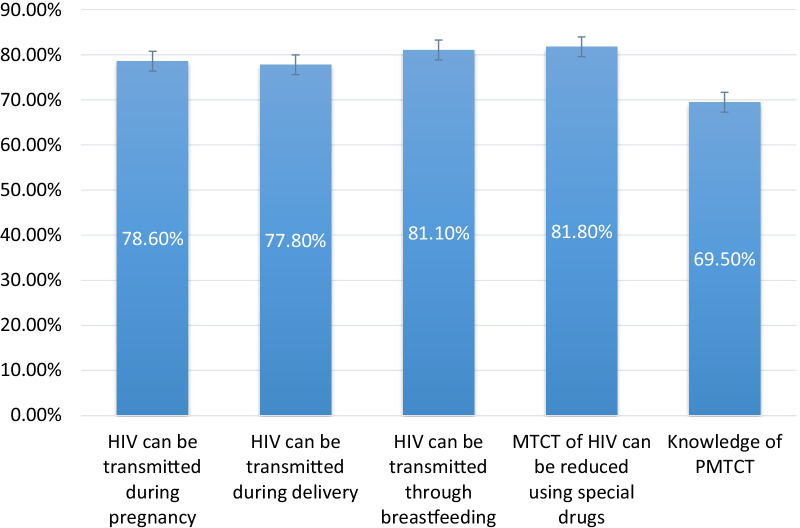
Knowledge of PMTCT among married women: Evidence from 24 SSA countries DHS

As shown in Fig. 1, the pooled results indicate that the overall knowledge of PMTCT among married women was 69.5%. About 78.6%, 77.9% and 81.2% of married women knew that HIV could be transmitted during pregnancy, delivery, and breastfeeding, respectively. Moreover, approximately 70.4% of married women knew all three aforementioned means of HIV transmission. About 81.8% of married women knew that taking special drugs could reduce the risk of mother-to-child transmission of HIV (Fig. 1).

### Knowledge of PMTCT across countries

**Fig. 2:**
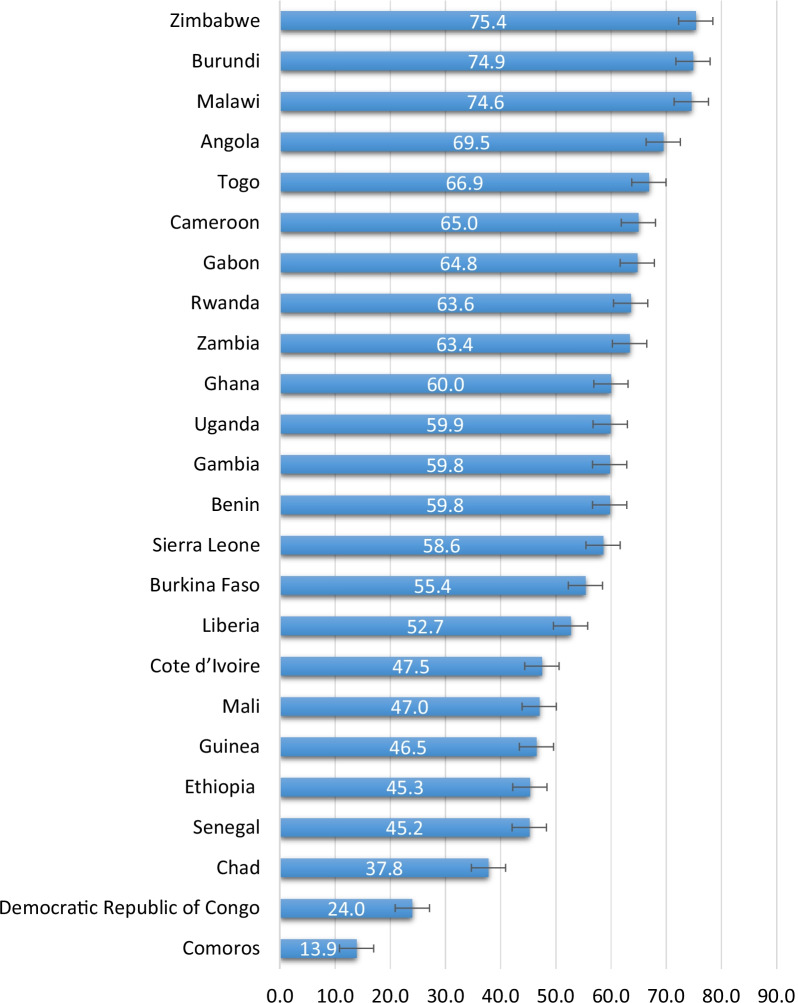
Knowledge of PMTCT among married women across countries: Evidence from 24 sub-Saharan African countries

Figure 2 shows variations of PMTCT knowledge across studied countries with the lowest coverage in Comoros (13.9%), Congo Democratic Republic (24.0%) and Chad (37.8%), and highest coverage in Zimbabwe (75.4%), Malawi (74.6%) and Burundi (74.9%), respectively (Fig. 2).

### Knowledge of PMTCT across explanatory/control variables

As shown in Table 2, PMTCT knowledge varied across explanatory variables. The study shows, PMTCT knowledge varied from 62.1 to 73.2% among married women who had no decision-making power for all three parameters and those who had decision making power. Moreover, PMTCT knowledge among married women with manual occupations was 81.6%, while it was 46.8% among married women with agricultural occupation. Similarly, PMTCT knowledge varied from 43.6 to 83.6% among married women within the poorest and richest households, respectively (Table 2).

### Bivariate and Multivariable logistic regression results

As shown in Table 3, from the bivariate logistic regression analysis, factors such as women’s decision-making power, women’s age, women’s educational level, husband educational level, women’s occupation, wealth index, parity, barriers to healthcare access, place of residence, mass media exposure had significant association with knowledge of PMTCT among married women. However, in multivariable logistic regression analysis, women’s decision-making power, women’s occupation type, wealth index and parity were significantly associated with knowledge of PMTCT.

**Table 3:** Bivariate and multivariable results for association between women’s decision-making power and knowledge of PMTCT: Evidence from 24 SSA countries DHS

Variables	Model I		Model II	
	cOR [95% CI]	*P*-value	aOR [95% CI]	*P*-value
*Decision making*				
No	Ref		Ref	
Yes	1.66 (1.39–1.99)	**< 0.001**	1.49 (1.24–1.78)	**< 0.001**
*Age in years*				
15–19	Ref		Ref	
20–24	1.50 (1.08–2.10)	**0.016**	1.16 (0.81–1.67)	0.400
25–29	2.04 (1.42–2.93)	**< 0.001**	1.48 (0.97–2.25)	0.065
30–34	2.06 (1.43–2.99)	**< 0.001**	1.52 (0.98–2.35)	0.059
35–39	1.80 (1.17–2.77)	**0.007**	1.34 (0.83–2.14)	0.221
40–44	1.66 (1.12–2.45)	**0.011**	1.32 (0.81–2.14)	0.257
45–49	1.48 (0.94–2.32)	0.088	1.20 (0.71–2.04)	0.486
*Women’s educational level*				
No formal education	Ref		Ref	
Primary school	1.20 (0.95–1.51)	0.118	0.83 (0.65–1.06)	0.144
Secondary school	2.53 (1.99–3.21)	**< 0.001**	1.04 (0.80–1.37)	0.726
Higher	2.72 (1.51–4.88)	**0.001**	0.75 (0.37–1.49)	0.418
*Husband’s educational level*				
No formal education	Ref		Ref	
Primary school	0.77 (0.58–1.02)	0.076	0.88 (0.67–1.14)	0.352
Secondary school	1.69 (1.34–2.13)	**< 0.001**	1.05 (0.82–1.36)	0.651
Higher	2.45 (1.61–3.73)	**< 0.001**	0.95 (0.60–1.50)	0.852
*Women’s occupation*				
Not working	Ref		Ref	
Professional or technical or managerial	1.33 (0.87–2.03)	0.180	0.89 (0.56–1.40)	0.621
Agricultural	0.34 (0.26–0.44)	**< 0.001**	0.88 (0.65–1.19)	0.436
Manual	1.74 (1.07–2.84)	**0.025**	1.68 (1.04–2.72)	**0.034**
Others	1.15 (0.91–1.46)	0.220	1.04 (0.82–1.32)	0.699
*Wealth index*				
Poorest	Ref		Ref	
Poor	1.11 (0.85–1.46)	0.407	0.98 (0.74–1.30)	0.916
Middle	2.64 (1.99–3.52)	**< 0.001**	1.79 (1.23–2.61)	**0.002**
Rich	4.19 (3.03–5.81)	**< 0.001**	2.54 (1.61–3.99)	**< 0.001**
Richest	6.59 (4.64–9.36)	**< 0.001**	3.93 (2.43–6.34)	**< 0.001**
*Parity*				
Zero	Ref		Ref	
1–2	1.87 (1.17–3.00)	**0.009**	1.82 (1.07–3.11)	**0.027**
3–4	1.75 (1.10–2.78)	**0.018**	1.59 (0.93–2.72)	0.085
5+	1.39 (0.87–2.22)	0.166	1.50 (0.84–2.66)	0.161
*Barriers to access healthcare*				
No	Ref		Ref	
Yes	0.71 (0.57–0.87)	**0.001**	0.92 (0.74–1.13)	0.442
*Place of residence*				
Urban	Ref		Ref	
Rural	0.28 (0.23–0.36)	**< 0.001**	0.76 (0.56–1.02)	0.075
*Mass media exposure*				
No	Ref		Ref	
Yes	2.38 (1.93–2.93)	**< 0.001**	0.96 (0.76–1.21)	0.740

Bold values indicate differentiate the variables

Ref = reference category

Specifically, the study shows higher odds of PMTCT knowledge among married women who had decision-making power (aOR = 1.49, 95% CI; 1.24–1.78) compared to married women who had no decision-making power. Moreover, we found higher odds of PMTCT knowledge among married women with manual occupation types (aOR = 1.68, 95% CI; 1.04–2.72) compared to married women who were not working. We found that married women from the richest households had higher odds of PMTCT knowledge (aOR = 3.93, 95% CI; 2.43–6.34) compared to married women in the poorest households. Finally, higher odds of knowledge of PMTCT were seen among married women with 1–2 parity history (aOR = 1.82, 95% CI; 1.07–3.11) compared to married women with no parity (Table 3).

The country-specific result showed, higher odds of PMTCT knowledge among married women who had decision-making power as compared to those who had no decision making power in Angola, Burkina Faso, Benin, Congo Democratic Republic, Cameroon, Ethiopia, Gabon, Ghana, Gambia, Guinea, Malawi, Rwanda, Uganda, and Zimbabwe (Table 4).

**Table 4:** Bivariate and multivariable results for association between women’s decision-making power and knowledge of PMTCT by country: evidence from 24 SSA countries DHS

Country	cOR (95% CI)	*P*-value	aOR (95% CI)	*P*-value
Angola	1.40 ((1.25–1.58)	**< 0.001**	1.30 (1.15–1.47)	**< 0.001**
Burkina Faso	1.48 (1.31–1.66)	**< 0.001**	1.30 (1.15–1.48)	**< 0.001**
Benin	1.46 (1.29–1.67)	**< 0.001**	1.42 (1.24–1.62)	**< 0.001**
Burundi	1.06 (0.96–1.16)	0.232	1.09 (0.99–1.20)	0.077
Congo Democratic Republic	1.26 (1.13–1.40)	**< 0.001**	1.22 (1.10–1.36)	**< 0.001**
Cote d’lvoire	1.34 (1.17–1.53)	**< 0.001**	1.08 (0.92–1.23)	0.372
Cameroon	1.90 (1.71–2.10)	**< 0.001**	1.52 (1.35–1.70)	**< 0.001**
Ethiopia	1.69 (1.52–1.87)	**< 0.001**	1.42 (1.27–1.59)	**< 0.001**
Gabon	1.25 (1.09–1.42)	**0.001**	1.16 (1.01–1.34)	**0.032**
Ghana	1.38 (1.22–1.56)	**< 0.001**	1.24 (1.09–1.41)	**0.001**
Gambia	1.92 (1.72–2.14)	**< 0.001**	1.85 (1.65–2.07)	**< 0.001**
Guinea	1.26 (1.11–1.42)	**< 0.001**	1.35 (1.18–1.54)	**< 0.001**
Comoros	1.04 (0.82–1.31)	0.730	0.94 (0.74–1.19)	0.631
Liberia	0.98 (0.85–1.13)	0.818	0.98 (0.84–1.14)	0.844
Mali	0.89 (0.75–1.06)	0.212	0.87 (0.73–1.03)	0.120
Malawi	1.15 (1.07–1.24)	**< 0.001**	1.09 (1.01–1.18)	**0.017**
Rwanda	1.09 (0.98–1.21)	0.100	1.13 (1.01–1.25)	**0.026**
Sierra Leone	1.05 (0.96–1.16)	0.250	0.99 (0.90–1.10)	0.958
Senegal	0.98 (0.87–1.11)	0.822	0.96 (0.85–1.09)	0.592
Chad	1.17 (0.95–1.43)	0.120	0.96 (0.76–1.20)	0.729
Togo	0.96 (0.85–1.09)	0.591	0.98 (0.86–1.12)	0.870
Uganda	1.10 (1.02–1.18)	**0.012**	1.09 (1.01–1.18)	**0.025**
Zambia	1.15 (1.04–1.27)	**0.003**	1.10 (0.99–1.22)	0.054
Zimbabwe	1.37 (1.20–1.57)	**< 0.001**	1.30 (1.14–1.49)	**< 0.001**

Bold values indicate differentiate the variables

## Discussion

In this study, knowledge of PMTCT and its association with women’s decision-making power was examined using nationally representative data from 24 sub-Saharan African countries. The pooled results show that them majority of married women had knowledge of PMTCT. This finding is slightly lower than that of a prior study in BosomemFreho District, Ashanti region of Ghana that showed the coverage of knowledge of PMTCT among women was 77% [32]. However, another study in Ghana (10%) [10] and other studies in Ethiopia (34.9%) [15], Assosa town, Northwest Ethiopia (17.4%) [33], Mecha district, Ethiopia (22.4%) [8], Cameroon (23.7%) [11] and Tanzania (46%) [9], documented lower coverage of PMTCT knowledge.

We found that women’s decision-making power had a significant positive association with knowledge of PMTCT among married women. Unlike women who had decision-making capacity, women who had no decision-making power about their own health usually have poor access to healthcare services [34]. This might be related to needing permission from their husband and financial dependency on their husband [34]. Women’s decision-making power has also been linked with education [35, 36]. Prior studies in different areas of Ethiopia [8, 12, 13, 15] and Tanzania [37] have found higher odds of PMTCT knowledge among women with higher levels of education. Education has a considerable role in improving and creating a good opportunity for information, occupation, and income [38]. Moreover, education facilitates the acquisition of knowledge about maternal health services and enables women to seek and utilize healthcare services [39, 40]. Women who utilize healthcare services such as ANC and health facility delivery are more likely to have PMTCT knowledge [12].

Moreover, the current study shows an association between women’s occupation and knowledge of PMTCT. Comparable findings are reported in Ethiopia [15]. This might be related to employed women having better income and economic status [40]. This might also be linked to the association between employment and maternal health services [41]. Employment is a good mechanism for income and improving women’s economic status, which is linked with healthcare service utilization [41].

In this study, higher odds of PMTCT knowledge among wealthier women were observed compared to the poorest women. Consistent findings were reported in Kenya [14] and Ethiopia [15]. The plausible reason might be due to women in lower socioeconomic status having less access to healthcare services or health facilities (potential site for health acquire information), because of financial barriers [42, 43]. That in turn makes them to not utilize maternal health services such as ANC [44–46]. The other mechanism for better knowledge of PMTCT among married women with higher economic status might be related to individuals in the higher socioeconomic class possibly having better media exposure and educational opportunities, which then increase their level of knowledge [47, 48]. This is confirmed by a prior study in Ethiopia that showed higher odds of PMTCT knowledge among women who had media exposure compared to women who had no media exposure [15].

Finally, we found associations between parity and knowledge of PMTCT, with higher knowledge of PMTCT knowledge among married women with history of parity as compared to married women with no parity history. Our finding is comparable with prior studies in South Ethiopia [12]. This could be related to prior experience of visiting health facilities and exposure to health information [49, 50].

Examining the influence of women’s decision-making power on PMTCT knowledge using nationally representative and large samples from several countries increased the strength of the paper. Therefore, we anticipate that the findings from this study would contribute to addressing the current knowledge and help in designing new policies and strategies to strengthen PMTCT programmes and control the HIV pandemic, especially in sub-Saharan African countries. Nonetheless, the findings should be seen in the perspective of the following limitations. First, though we included most countries, still there are few sub-Saharan African countries that were excluded from the analysis because of the inclusion criteria; as a result, generalizing the findings to all sub-Saharan African countries may not be possible. Second, due to the cross-sectional nature of DHS data, establishing a cause-effect relationship may not be possible. Finally, the survey participants’ self-reported data may be affected by recall bias.

## Conclusion

Women’s decision-making power had positive association with PMTCT knowledge. Moreover, we found that women’s occupation, wealth index and parity were positively associated with knowledge of PMTCT among married women. Therefore, to increase the knowledge of PMTCT, especially in countries with lower knowledge of PMTCT, policy makers, programme planners, and other stakeholders working on HIV control need to innovate about ways that can enhance women’s decision-making power. Furthermore, special focus on creating employment opportunities and economic empowerment for married women could be considered.

## Data Availability

Data for this study were sourced from Demographic and Health surveys (DHS) and available here: http://dhsprogram.com/data/available-datasets.cfm.
